# Bilateral Foot Drop as the Presenting Manifestation of Systemic Lupus Erythematosus-Associated Mononeuritis Multiplex: A Case Report

**DOI:** 10.1159/crn/abyag002

**Published:** 2026-08-11

**Authors:** Fadel Alqatati, Mohammad Ghabashneh, Muntasir Alanasweh, Mohamad Wajeh Dulli, Mustafa Abufouda, Hasan Hani Awad, Muhammad Naeem

**Affiliations:** a Department of Internal Medicine, Hamad Medical Corporation, Doha, Qatar; b Department of Neurology, Hamad Medical Corporation, Doha, Qatar; c Clinical Imaging Department, Hamad Medical Corporation, Doha, Qatar

**Keywords:** Systemic lupus erythematosus, Bilateral foot drop, Mononeuritis multiplex, Vasculitic neuropathy, Case report

## Abstract

**Introduction:**

Peripheral nervous system involvement in systemic lupus erythematosus (SLE) is uncommon, and mononeuritis multiplex as the presenting neurological manifestation is particularly rare. Early recognition may be difficult when the initial clinical picture resembles focal compressive neuropathy.

**Case Presentation:**

A 31-year-old previously healthy woman developed acute right foot numbness and foot drop after right knee pain and earlier nonspecific arthralgia. Initial assessments favored peroneal neurapraxia. Over the following 3 weeks, her deficits progressed to persistent right foot drop followed by new left lower limb numbness and weakness, resulting in asymmetric bilateral foot drop. Electrophysiological studies demonstrated an asymmetric, non-length-dependent motor and sensory axonal neuropathy consistent with mononeuritis multiplex. Magnetic resonance imaging of the lumbosacral spine excluded a compressive lumbosacral explanation and focused ultrasound showed no compressive lesion involving the right common peroneal nerve. Serological evaluation revealed high-titer antinuclear antibody, positive anti-double-stranded DNA, anti-Ro, anti-ribosomal P antibodies, elevated inflammatory markers, and a positive lupus anticoagulant screen, with negative infectious screening and no evidence of active lupus nephritis. She was treated with intravenous methylprednisolone, intravenous immunoglobulin, hydroxychloroquine, and rituximab, followed by oral corticosteroid tapering and rehabilitation. Her pain improved early, distal motor function partially recovered, and she later demonstrated meaningful functional improvement with orthotic and rehabilitation support.

**Conclusion:**

This case suggests that SLE may initially present predominantly as mononeuritis multiplex, with evolving bilateral foot drop as the dominant neurological and function-threatening manifestation, through a suspected vasculitic mechanism, even when more typical renal, serosal, or major mucocutaneous features are absent at neurological presentation. In patients with progressive or sequential foot drop, especially when the presentation becomes bilateral or multifocal, prompt electrodiagnostic testing and evaluation for systemic autoimmune disease are essential to avoid diagnostic delay and allow timely immunotherapy.

## Plain Language Summary

Foot drop means difficulty lifting the front part of the foot, which can cause tripping or an abnormal walking pattern. It is often caused by pressure on a nerve near the knee or by a problem in the lower back. This case describes a 31-year-old woman who first developed numbness and weakness in her right foot. At first, her symptoms looked similar to a common nerve compression problem. Over the next few weeks, she developed symptoms in both legs. Nerve tests showed that several separate nerves were affected. This pattern is called mononeuritis multiplex. Blood tests also supported a new diagnosis of systemic lupus erythematosus, an autoimmune disease in which the immune system can attack the body’s own tissues. In this patient, the nerve problem was considered most likely related to lupus-associated inflammation of the small blood vessels that supply the nerves, although this was not confirmed by nerve biopsy. She was treated with medicines to reduce immune system activity, including steroids, intravenous immunoglobulin, hydroxychloroquine, and rituximab. She also received physiotherapy and ankle-foot supports to improve walking. Her pain and movement improved, although some weakness remained during follow-up. This case highlights that worsening or bilateral foot drop should not always be assumed to be due to simple nerve compression. When symptoms progress or affect both sides, doctors should consider inflammatory and autoimmune causes and arrange appropriate nerve and blood tests.

## Introduction

Peripheral nervous system (PNS) disease is an uncommon component of systemic lupus erythematosus (SLE). In a large international inception cohort, PNS disease occurred in 139 of 1,827 patients (7.6%); among PNS events, mononeuropathy represented 27.3% [1]. A separate retrospective cohort of 1,224 patients reported PNS involvement in 6.9%, with multiple mononeuritis accounting for 8 of 97 PNS events (8.2%), corresponding to approximately 0.7% of the whole cohort [2]. These data emphasize that multiple mononeuropathy is recognized but uncommon, and it may be overlooked when the initial syndrome appears focal [1–5].

From a neurological perspective, the principal challenge is diagnostic. Acute foot drop is more often attributed to common peroneal compression or lumbosacral radiculopathy, whereas inflammatory or vasculitic neuropathy requires broader systemic and neurophysiological evaluation. Current recommendations support glucocorticoids, hydroxychloroquine, and escalation to immunosuppressive therapy for active inflammatory organ-threatening lupus, while the vasculitic neuropathy literature emphasizes early recognition and treatment to reduce irreversible axonal injury [6, 7].

We report a young woman whose illness began as apparently isolated right foot drop and evolved over the following weeks into asymmetric bilateral foot drop with electrophysiological evidence of mononeuritis multiplex, ultimately leading to the diagnosis of probable SLE-associated mononeuritis multiplex, likely mediated by small vessel vasculitic injury to the vasa nervorum. The main clinical value of this case lies not in claiming an unprecedented phenomenon, but in illustrating a diagnostically misleading early phenotype that initially resembled focal peroneal neuropathy. This case report was prepared in accordance with the CARE reporting guideline, and the completed CARE checklist is provided as online supplementary Material (for all online suppl. material, see https://doi.org/10.1159/crn/abyag002).

## Case Report

A 31-year-old previously healthy Filipino woman developed right knee pain followed by numbness and paresthesia below the right knee, which progressed over several days to right foot drop. An earlier outpatient assessment had documented arthralgia involving the knees, elbows, and small joints of the hands and feet, with stiffness improving during the day; however, at neurological admission, the active musculoskeletal findings were mainly periarticular right knee and ankle/heel-malleolar pain, mild swelling, and tenderness. She had no known history of diabetes mellitus, autoimmune disease, chronic hypertension, or previous neurological disorder. She had a remote history of conservatively managed lumbar vertebral fractures after a road traffic accident without residual neurological deficits and a history of treated latent tuberculosis.

At the first medical evaluation for the neurological symptoms, she denied recent infection, vaccination, back pain, trauma, bowel or bladder dysfunction, and upper limb symptoms. The pain was periarticular, involving mainly the right knee and ankle/heel-malleolar region, with local tenderness and mild swelling, rather than electric, lancinating, or radiating neuritic pain. There was no focal tenderness along the course of the common peroneal nerve. Neurological examination showed preserved mental status, intact cranial nerves, and normal upper limbs. In the right lower limb, weakness predominantly affected ankle dorsiflexion with relatively preserved plantar flexion, accompanied by sensory disturbance below the knee. She was initially treated as having right common peroneal neurapraxia and discharged with outpatient neurological follow-up. During the following week, she continued to experience right foot weakness with persistent lateral leg numbness. Neurology assessment showed weakness of dorsiflexion and eversion, approximately 3/5, with reduced sensation over the lateral aspect of the right leg. Because the presentation still appeared compatible with focal peroneal neuropathy, nerve conduction studies were arranged.

Over the next 2 weeks, her symptoms progressed rather than improved. She developed recurrent right foot pain with mild ankle swelling and increasing difficulty bearing weight. Shortly afterward, new symptoms appeared in the left lower limb, including numbness and distal weakness, raising concern for a non-compressive multifocal neuropathic process. The chronological progression of symptoms, investigations, treatment, and follow-up is summarized in Table 1. At this stage, she denied visual symptoms, diplopia, dysarthria, facial weakness, upper limb weakness, saddle anesthesia, bowel or bladder dysfunction, oral ulcers, Raynaud phenomenon, genital ulcers, digital ulcers, or significant constitutional symptoms. She reported habitual leg crossing.

**Table 1.** Clinical timeline of symptom progression, diagnostic evaluation, and therapeutic intervention

Time from symptom onset	Clinical event	Key findings	Investigations	Management	Outcome
Initial few days	Symptom onset	Right knee pain/arthralgia followed by numbness and paresthesia below the right knee; evolving right foot drop	Initial clinical assessment	Analgesia/supportive treatment	Weakness persisted
First presentation	ED/early neurology assessment	Right foot drop with distal sensory disturbance; no back pain, sphincter symptoms, upper limb symptoms, recent infection, or vaccination	Neurological examination; outpatient NCS planned	Initially considered right common peroneal neurapraxia	No sustained improvement
Following 2–3 weeks	Progression	Persistent right foot drop and new left lower limb numbness/distal weakness; asymmetric bilateral foot drop	Admission for inpatient workup	Neurology and medicine assessment	Multifocal neuropathy considered
Inpatient workup	Autoimmune/inflammatory assessment	High-titer ANA, positive anti-dsDNA, anti-Ro and anti-ribosomal P; elevated ESR/CRP; single positive lupus anticoagulant screen; normal complements; bland urine	Autoimmune, infectious, metabolic, and renal assessment	Rheumatology consultation	New diagnosis of SLE with probable inflammatory neuropathy
Inpatient workup	Electrophysiological confirmation	Asymmetric, non-length-dependent axonal motor and sensory neuropathy; peroneal-predominant with tibial motor involvement	Formal EMG/NCS.	Interpreted as mononeuritis multiplex; see Table 2	Compressive mononeuropathy alone became less likely
Inpatient workup	Exclusion of structural causes	MRI lumbosacral spine: old L5 fracture and minor disc bulges without canal/foraminal compromise; right peroneal nerve ultrasound without compressive lesion; MRA without large vessel vasculitis	MRI spine, focused ultrasound, MRA aorta/major branches, TTE.	Supportive care and systemic evaluation	Radiculopathy and focal compression were not supported
Week 4–5	Acute immunotherapy	Progressive, function-threatening bilateral distal weakness	Clinical monitoring	IV methylprednisolone 500 mg daily for 5 days; IVIG 25 g daily for 4 days; hydroxychloroquine 200 mg daily	Pain improved and early toe movement returned
After infectious disease clearance	Escalation therapy	History of treated latent TB; no active infection	ID assessment; HBV/HCV/HIV screening	Rituximab 1 g IV, with second dose 2 weeks later	No major infusion complication; continued recovery
Follow-up	Rehabilitation and outcome	Residual distal weakness with functional improvement	Rehab assessments and inflammatory markers	Physiotherapy and dynamic ankle-foot orthoses	Independent transfers/self-care and improved ambulation

AFO, ankle-foot orthosis; ANA, antinuclear antibody; anti-dsDNA, anti-double-stranded DNA; CRP, C-reactive protein; ED, emergency department; EMG, electromyography; ESR, erythrocyte sedimentation rate; IVIG, intravenous immunoglobulin; MRA, magnetic resonance angiography; NCS, nerve conduction studies; SLE, systemic lupus erythematosus; TTE, transthoracic echocardiography.

On admission, serial neurological examinations demonstrated a distal, asymmetric, lower limb-predominant neuropathy. Mental status and cranial nerves remained normal. Upper limb examination was normal, and proximal lower limb strength was preserved. Distally, ankle dorsiflexion was reduced bilaterally, more severe on the right (approximately 3/5) than the left (approximately 4/5). Plantar flexion was mildly reduced on the right and near normal on the left. Ankle eversion and inversion were documented as weak bilaterally, approximately 3/5**.** Sensation to light touch and pinprick was reduced over the dorsum of both feet and along the lateral aspect of the left leg below the knee. Reflexes were preserved or only mildly reduced, plantar responses were downgoing, coordination was intact, and gait was steppage in character. There was no upper motor neuron pattern, no cranial nerve involvement, and no sphincter disturbance.

Laboratory evaluation showed mild normocytic anemia, with hemoglobin 11.6 g/dL, and elevated inflammatory markers, including erythrocyte sedimentation rate 64 mm/h (reference range, 2–37 mm/h) and C-reactive protein 10.7 mg/L (reference range, 0–5 mg/L). Autoimmune serology showed strongly positive antinuclear antibody at 1:1,280 with a homogeneous pattern, anti-double-stranded DNA (anti-dsDNA) antibody 40 IU/mL, anti-Ro antibody 78 U/mL, and anti-ribosomal P antibody 48 U/mL. Lupus anticoagulant screening was positive on a single set, with a ratio of 1.77 (reference range, 1.01–1.41), although subsequent antiphospholipid antibody testing did not support persistent antiphospholipid syndrome. Complement levels were initially preserved, with C3 1.02 g/L and C4 0.25 g/L, whereas follow-up showed low C3 of 0.65 g/L with improved inflammatory markers and anti-dsDNA antibody level. Rheumatoid factor, antineutrophil cytoplasmic antibodies, and cryoglobulins were negative. Glycated hemoglobin, vitamin B12, folate, and thyroid function tests were normal. Hepatitis B, hepatitis C, and human immunodeficiency virus screening were negative. Renal function was preserved, and urinalysis showed bland sediment without significant proteinuria or hematuria, with no evidence of active lupus nephritis.

Electrophysiological testing was the key diagnostic investigation. An initial private nerve conduction study suggested severe right and mild left common peroneal axonal neuropathy. Formal inpatient electromyography (EMG) and nerve conduction studies later demonstrated an asymmetric, non-length-dependent motor and sensory axonal neuropathy of differing acuity, consistent with mononeuritis multiplex (Table 2). Findings included markedly reduced bilateral fibular motor amplitudes, reduced right tibial motor amplitude, absent right superficial fibular sensory response, abnormal right sural sensory response, absent fibular F-wave responses bilaterally, and active denervation with reduced recruitment in the right tibialis anterior. On review of the right sural tracing, the visible deflection was very low in amplitude and not reproducible on repeat stimulation; therefore, it was interpreted as markedly reduced/non-reproducible rather than a reliable normal sensory nerve action potential. The unusually high calculated left deep fibular conduction velocity to tibialis anterior was interpreted cautiously because the segment was short and waveform morphologies were not identical. Representative tibial motor traces are shown in Figure 1.

**Table 2.** Consolidated electrodiagnostic findings from nerve conduction studies and needle EMG

Study type	Nerve/muscle tested	Side	Key abnormality	Interpretation	Comment/limitation
Motor NCS	Fibular motor to extensor digitorum brevis (EDB)	Bilateral	Markedly reduced CMAP amplitudes: left ankle 0.53 mV; right ankle 0.24 mV, with further low proximal amplitudes	Bilateral fibular motor axonal involvement, right worse than left	Conduction velocities were not in a demyelinating range; low amplitudes limit interpretation of amplitude drop
Motor NCS	Deep fibular branch to tibialis anterior	Bilateral	Left amplitudes preserved with reported very high calculated velocity (74 m/s); right amplitudes lower with velocity 49 m/s	Supports asymmetric fibular involvement but not primary demyelination	The left calculated velocity and dissimilar waveform morphology were interpreted cautiously, likely reflecting short-segment/technical measurement factors rather than a biologically meaningful supernormal velocity
Motor NCS	Tibial motor to abductor hallucis brevis (AHB)	Bilateral	Left tibial amplitude fell from 8.6 mV at ankle to 3.2 mV at knee; right tibial response low at ankle (1.51 mV) and absent at knee	Additional tibial motor involvement; apparent conduction block/pseudo-block pattern	In vasculitic neuropathy, apparent conduction block can reflect focal axonal conduction failure; serial studies would be required to confirm true demyelinating conduction block
Motor NCS	Median and ulnar motor studies	Bilateral	Preserved distal amplitudes and velocities	Upper limb motor nerves relatively spared	Supports non-length-dependent/asymmetric rather than diffuse generalized neuropathy
Sensory NCS	Superficial fibular sensory	Bilateral	Right superficial fibular sensory response absent; left superficial fibular amplitude borderline/low-normal	Sensory involvement in fibular distribution	Contributes to sensorimotor mononeuritis multiplex pattern
Sensory NCS	Sural sensory	Bilateral	Left sural response preserved. Right sural trace showed a very low amplitude deflection (P-P approximately 2 microV, below normal) that was not reproduced on repeat stimulation	Right sural response interpreted as markedly reduced/non-reproducible rather than a robust normal SNAP.	This clarifies the discrepancy between the tabulated NR entry and the visible trace; the apparent deflection was not considered a reliable normal sensory potential
Sensory NCS	Median, radial, and ulnar sensory studies	Upper limbs	Responses preserved	Upper limb sensory nerves relatively spared	Further supports multifocal lower limb-predominant process
Needle EMG	Tibialis anterior	Right	Active denervation with 3+ fibrillation potentials/positive sharp waves and reduced recruitment/interference pattern	Active axonal injury in the right deep fibular distribution	Consistent with clinical right-predominant foot drop
Needle EMG	Gastrocnemius	Right	No active denervation; reduced recruitment/interference pattern	Tibial/S1-2 distribution less severely affected than fibular distribution	Helps show patchy involvement
Needle EMG	Tibialis anterior	Left	Reported “no activation,” without documented pain/cooperation details	Limited left TA activation prevents reliable grading of recruitment on that side	Not interpreted as proof of more severe left-sided disease; the clinical examination remained right-predominant early
Needle EMG	Biceps femoris short head	Not tested	Not assessed	Localization limitation	Because this muscle was not sampled, EMG could not fully distinguish sciatic neuropathy from multiple peripheral nerve lesions around the knee; clinical course and NCS distribution favored mononeuritis multiplex

CMAP, compound muscle action potential; EDB, extensor digitorum brevis; EMG, electromyography; NCS, nerve conduction study; NR, no reproducible response; SNAP, sensory nerve action potential.

**Fig. 1.**
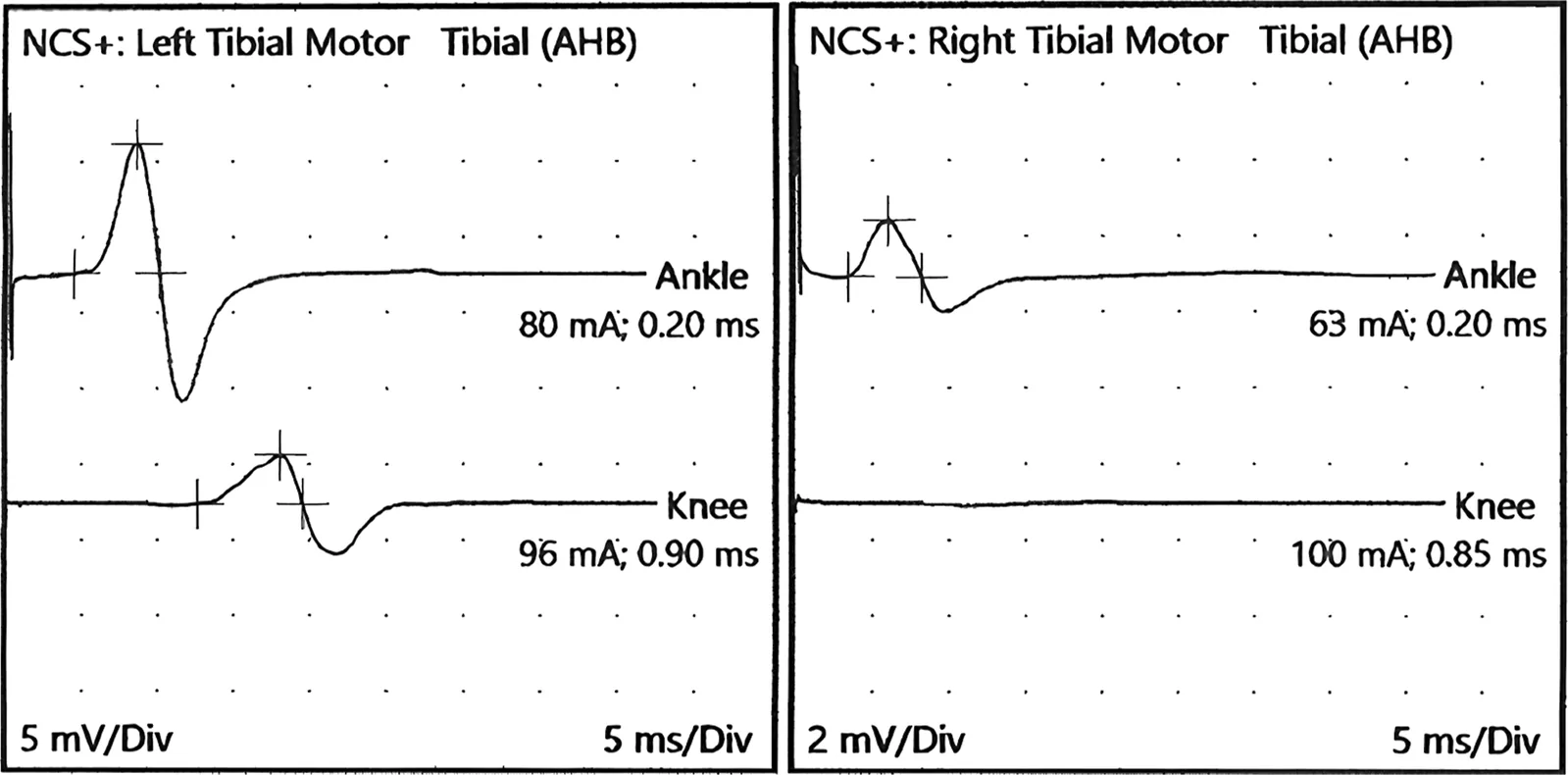
Representative left and right tibial motor nerve conduction study traces. The left tibial response shows amplitude reduction between ankle and knee stimulation, whereas the right tibial response is low at the ankle and absent/non-reproducible proximally. In the clinical context of asymmetric axonal sensorimotor neuropathy, these traces were interpreted as tibial motor involvement with possible pseudo-conduction block rather than definitive primary demyelinating conduction block.

Needle EMG did not include the biceps femoris short head. This limits precise electrodiagnostic localization between sciatic neuropathy and multiple peripheral nerve lesions around the knee. In addition, the left tibialis anterior was reported as “no activation”; because the report did not document pain, cooperation, or technical difficulty, this was treated as a study limitation rather than evidence that the left side was more severely affected than the clinically worse right side.

Magnetic resonance imaging (MRI) of the lumbosacral spine showed remote vertebral fracture deformity with minor degenerative disc disease but no significant spinal canal stenosis or nerve-root compression sufficient to explain bilateral foot drop. Focused ultrasound of the right common peroneal nerve showed no focal thickening or compressive lesion. Magnetic resonance angiography (MRA) of the thoracic and abdominal aorta and major branches showed no large vessel vasculitis and no renal artery stenosis. Chest radiography was unremarkable, and transthoracic echocardiography showed normal cardiac function without pericardial effusion.

The major differential diagnoses considered included bilateral compressive peroneal neuropathy, lumbosacral radiculopathy, hereditary neuropathy with liability to pressure palsies, inflammatory demyelinating neuropathies including acute Guillain-Barré syndrome and chronic variants such as multifocal acquired demyelinating sensory and motor neuropathy (MADSAM), diabetic neuropathy, infectious neuropathy, and immune-mediated mononeuritis multiplex. The progressive bilateral course, asymmetric axonal neurophysiology, inflammatory serology, and exclusion of structural, diabetic, and infectious causes strongly favored probable SLE-associated mononeuritis multiplex, likely vasculitic, rather than isolated entrapment neuropathy. Because no nerve or muscle biopsy was performed, the diagnosis was regarded as probable rather than histologically confirmed vasculitic neuropathy.

Because of progressive and function-threatening neurological deficits, treatment was initiated with intravenous methylprednisolone 500 mg daily for 5 days, followed by intravenous immunoglobulin 25 g daily for 4 days. Hydroxychloroquine 200 mg daily was added for long-term SLE management. After infectious diseases clearance because of previously treated latent tuberculosis, rituximab therapy was initiated, with the first dose administered before discharge and the second dose given 2 weeks later. During IVIG infusion, she developed transient sinus tachycardia without hypotension, hypoxemia, rash, or bronchospasm. The infusion was temporarily stopped and later resumed at a slower rate with good tolerance. A similar transient tachycardia occurred during rituximab infusion and was managed conservatively without interruption of treatment.

Neurological improvement began during admission, with reduction in pain and partial return of toe movement. She was discharged on a tapering course of oral prednisolone, hydroxychloroquine, calcium, vitamin D supplementation, cotrimoxazole prophylaxis, gastric protection, and outpatient physiotherapy with orthotic support.

At early outpatient rehabilitation review, distal weakness remained marked, and she required a wheelchair or quad cane for short distances, with ankle dorsiflexion and extensor hallucis longus strength documented as 1/5 bilaterally. With continued immunotherapy, orthotic support, and structured rehabilitation, she subsequently demonstrated substantial functional recovery. At discharge from the adult day-care rehabilitation program, sensation was documented as intact, lower limb strength had improved to approximately 4/5 bilaterally, and she was independent in transfers and modified independent in ambulation and stair negotiation using an ankle-foot orthosis. Objective rehabilitation measures improved, including the 6-min walk distance from 210 m to 330 m, Dynamic Gait Index from 17/24 to 21/24, functional ambulatory category from 4/5 to 5/5, and Functional Independence Measure walking and stair scores to 7/7. Repeat inflammatory markers and anti-dsDNA antibody levels also improved, supporting clinical response to immunotherapy and rehabilitation.

## Discussion

The central feature of this case is that SLE became clinically apparent through an evolving peripheral nerve syndrome rather than through a more typical pattern of renal, serosal, mucocutaneous, or hematological disease. PNS disease occurs in only a minority of patients with SLE, and multiple mononeuropathy is distinctly less common than polyneuropathy [1–5]. This relative rarity contributes to diagnostic delay, particularly when the initial phenotype resembles focal entrapment neuropathy.

For neurologists, this case is educational because foot drop usually triggers a focused search for common peroneal compression or lumbosacral radiculopathy. In this patient, the tempo of progression was a crucial clue: what first appeared to be unilateral focal neuropathy evolved over roughly 3 weeks into bilateral, asymmetric distal weakness with multifocal sensory loss. Structural studies did not show a relevant compressive lesion, and electrophysiology demonstrated an asymmetric, non-length-dependent axonal sensorimotor neuropathy. That pattern is characteristic of mononeuritis multiplex and fits well with suspected vasculitic injury to the vasa nervorum [3, 7].

The lupus attribution is also instructive. The patient had little overt systemic lupus activity at onset, preserved initial complement levels, and bland urine studies, all of which could have lowered suspicion for active autoimmune disease. However, the combination of high-titer antinuclear antibody, positive anti-dsDNA, anti-Ro, and anti-ribosomal P antibodies, elevated inflammatory markers, and a clinical neurophysiological pattern typical of mononeuritis multiplex strongly supported SLE-associated inflammatory neuropathy. The single positive lupus anticoagulant result suggested transient antiphospholipid serology, but there was no clinical thrombotic syndrome and persistence was not established.

The major competing explanations were bilateral compressive common peroneal neuropathies, lumbosacral radiculopathy, hereditary neuropathy with liability to pressure palsies, inflammatory demyelinating neuropathies, and other immune or infectious neuropathies. A compressive contribution could not be excluded completely because of her leg-crossing habit and periarticular pain. Nevertheless, the stepwise bilateral progression, the neurophysiological distribution, the inflammatory serology, and the absence of a compressive lesion on targeted imaging made a purely mechanical explanation less persuasive. Lumbosacral radiculopathy was weakened by preserved sphincter function, lack of major back pain, and spinal imaging that did not show explanatory root compression. Diabetic and infectious causes were not supported by laboratory testing.

MADSAM, also known as Lewis-Sumner syndrome, was an important immune neuropathy mimic because it can present with asymmetric multifocal sensorimotor deficits. However, MADSAM is classically a demyelinating neuropathy characterized electrophysiologically by segmental slowing, temporal dispersion, and conduction block, whereas vasculitic neuropathies are usually axonal and asymmetric [8]. In the present case, the dominant abnormalities were reduced motor and sensory amplitudes with active denervation, supporting an axonal mononeuritis multiplex pattern. At the same time, focal conduction block or pseudo-conduction block can occur in vasculitic neuropathy because of focal axonal conduction failure related to ischemic/infarctive nerve injury; therefore, the tibial motor amplitude drop was interpreted cautiously and not used alone as evidence of a primary demyelinating neuropathy [9].

Published literature confirms that SLE-associated mononeuritis multiplex is rare but recognized. Earlier reports have described severe mononeuritis multiplex in SLE, sometimes secondary to necrotizing vasculitis, and other reports have described lupus-related vasculitic neuropathy presenting with wrist drop, foot drop, or isolated mononeuropathy multiplex [10–12]. Accordingly, the novelty of this report does not rest on SLE-associated mononeuritis multiplex itself, which has been previously described, but on the diagnostic sequence: an adult patient whose early presentation closely resembled unilateral common peroneal neurapraxia before evolving into asymmetric bilateral foot drop, with electrodiagnostic features of axonal mononeuritis multiplex and pseudo-conduction block pitfalls. This sequence is clinically relevant because it identifies when clinicians should move beyond a focal compressive neuropathy framework and evaluate for systemic autoimmune inflammatory neuropathy. Earlier recognition is important because timely immunotherapy and rehabilitation may limit irreversible axonal injury, reduce long-term neurological disability, and improve functional recovery.

Management was appropriately multidisciplinary and pragmatic. Current EULAR recommendations support hydroxychloroquine for all suitable patients with SLE and escalation to glucocorticoids and immunosuppressive therapy for organ-threatening disease [6]. In the broader vasculitic neuropathy literature, early immunotherapy is emphasized because delay risks permanent axonal loss [7]. In this patient, the combination of high-dose corticosteroids, IVIG, rituximab, and rehabilitation was followed by clear symptomatic, functional, and laboratory improvement, which supports the treating teams’ interpretation of inflammatory mononeuritis multiplex with a suspected vasculitic mechanism.

This report has important limitations. No nerve or muscle biopsy was performed; therefore, vasculitic neuropathy could not be histopathologically confirmed, and the diagnosis is best regarded as probable SLE-associated mononeuritis multiplex with a suspected vasculitic mechanism. The biceps femoris short head was not sampled by needle EMG, limiting precise localization between sciatic neuropathy and multiple peripheral nerve lesions around the knee. In addition, absent voluntary activation of the left tibialis anterior limited recruitment assessment on that side and was not interpreted as evidence that the left side was clinically more severely affected than the right. Serial electrodiagnostic testing was not available to determine whether the apparent conduction block evolved into axonal loss. Despite these limitations, the overall clinical neurophysiological pattern, SLE serology, exclusion of structural and metabolic mimics, and clinical response to immunotherapy supported probable SLE-associated mononeuritis multiplex with a likely vasculitic mrchanism.

This case reinforces a practical diagnostic principle: progressive or stepwise bilateral foot drop, especially when accompanied by multifocal sensory loss and axonal mononeuritis multiplex on electrophysiology, should prompt urgent evaluation for systemic autoimmune inflammatory neuropathy even when the initial presentation appears compatible with focal peroneal neuropathy. Early recognition is important because timely immunotherapy and rehabilitation may limit irreversible axonal injury, reduce long-term neurological disability, and improve functional outcome.

## Conclusion

This case represents probable SLE-associated mononeuritis multiplex presenting predominantly as evolving bilateral foot drop, with clinical and electrodiagnostic features compatible with a suspected vasculitic mechanism. It underscores the importance of reconsidering apparently focal foot drop when the clinical course becomes progressive, multifocal, or bilateral. Early electrodiagnostic evaluation and systemic autoimmune assessment may allow timely immunotherapy and rehabilitation, potentially limiting irreversible axonal injury, long-term neurological disability, and functional impairment.

## Acknowledgments

The authors thank the neurology, rheumatology, infectious diseases, rehabilitation, and radiology teams involved in the patient’s care.

## Statement of Ethics

This study was performed in accordance with the Declaration of Helsinki. This human study was approved by Hamad Medical Corporation, approval MRC-04-25-1459. Written informed consent was obtained from the patient for publication of the details of her medical case and any accompanying images.

## Conflict of Interest Statement

The authors have no conflicts of interest to declare.

## Funding Sources

This study was not supported by any sponsor or funder.

## Author Contributions

Fadel Alqatati contributed to case conception, case reconstruction, manuscript drafting, and literature review. Mohammad Ghabashneh contributed to neurological interpretation and critical revision of the manuscript. Muntasir Alanasweh, Mohamad Wajeh Dulli, and Hasan Hani Awad contributed to clinical data collection, clinical interpretation, and manuscript revision. Mustafa Abufouda contributed to radiological review and figure selection. Muhammad Naeem supervised the work and critically revised the manuscript. All authors reviewed and approved the final manuscript.

## Data Availability

All data generated or analyzed during this case report are included in this article and its online supplementary material. Further inquiries can be directed to the corresponding author.
